# Activation of CXCR7 promotes endothelial repair and reduces the carotid atherosclerotic lesions through inhibition of pyroptosis signaling pathways

**DOI:** 10.1111/acel.13205

**Published:** 2020-07-27

**Authors:** Lisha Qiu, Min Zhang, Sheng Zhang, Yalin Tang, Yanyan Zhang, Congcong Li, Yi Wang, Li Jiang, Jialin C. Zheng

**Affiliations:** ^1^ Center for Neuroimmunology and Regenerative Therapy Shanghai Tenth People's Hospital affiliated to Tongji University School of Medicine Shanghai China; ^2^ Tongji University School of Medicine Shanghai China; ^3^ Division of Cardiology Tongren Hospital Shanghai Jiao Tong University School of Medicine Shanghai China; ^4^ Collaborative Innovation Center for Brain Science Tongji University Shanghai China; ^5^ Department of Pharmacology and Experimental Neurosciences Nebraska Medical Center University of Nebraska Medical Center Omaha NE USA

**Keywords:** atherosclerosis, CXCR7, human umbilical vein endothelial cells, NLRP3, ox‐LDL

## Abstract

Endothelial injuries, including cell pyroptosis, are ongoing inflammatory processes with key roles in atherosclerosis development. Our previous report showed that the chemokine CXCL12 and its receptor CXCR7 are associated with the proliferation and angiogenesis of endothelial cells. Nevertheless, the mechanism underlying these effects on atherosclerotic lesions, especially on endothelial dysfunction, remains unknown. Here, we demonstrated that CXCR7 was upregulated in human carotid atherosclerotic plaques, apolipoprotein E knockout (ApoE^−/−^) mice fed with a high‐fat diet (HFD), and oxidized lipopolysaccharide‐treated (ox‐LDL) human umbilical vein endothelial cells (HUVECs). Further, the activation of CXCR7 reversed ox‐LDL‐induced HUVEC dysfunction, such as migration, tube formation, and cell pyroptosis; all of these protective effects were alleviated by inhibition of CXCR7. The NOD‐like receptor family pyrin domain‐containing 3 (NLRP3) inflammasomes were also elevated in human carotid atherosclerotic plaques, ApoE^−/−^ mice fed with HFD, and ox‐LDL‐injured HUVECs by regulation of caspase‐1 and interleukin (IL)‐1β expression. The activation of CXCR7 by TC14012 led to a decrease in atherosclerotic lesions in ApoE^−/−^ mice fed with HFD. TC14012 also inhibited the expression of the NLRP3 inflammasome signaling pathway *in vivo*. In conclusion, our study suggests that CXCR7 plays an important role in regulating NLRP3 inflammasome‐modulated pyroptosis in HUVECs, providing a potential novel therapy for atherosclerosis.

## Graphical abstract

CXCR7 is upregulated in human carotid plaque tissues, HFD‐treated ApoE^−/−^ mouse aortas, and ox‐LDL‐treated HUVECs. Activation of CXCR7 alleviates ox‐LDL‐induced endothelial injury and cell pyroptosis. Activation of CXCR7 reduces atherosclerosis progression by mitigating the pyroptosis pathway in vivo.

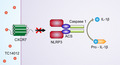

## 1 INTRODUCTION

Atherosclerosis is a chronic inflammatory disease and the dominant pathology of diverse cardiovascular diseases (GBD 2016 Lifetime Risk of Stroke Collaborators, 1990; Pourcet & Staels, 2018). It occurs mainly at lesion‐prone areas of large‐ and medium‐sized artery and manifests with oxidized low‐density lipoprotein (ox‐LDL) and accumulated in the intima of arteries (Kattoor, Kanuri, & Mehta, 2018; Ruiz‐Leon, Lapuente, Estruch, & Casas, 2019; Song, 2018; Wang, 2019). This may cause intima‐media thickening and lumenal stenosis and further give rise to several adverse vascular events, like myocardial infarction, coronary disease, stroke, or peripheral artery disease (Shah, Bajaj, Virk, Bikkina, & Shamoon, 2015).

As the most important component of intima, endothelial cell (EC) lining of blood vessels forms a protective barrier against endogenous and exogenous stress conditions (Beldman et al., 2019). Previous reports have shown that endothelial dysfunction is a key initial stage for the development of atherosclerosis (Lane‐Cordova, Kershaw, Liu, Herrington, & Lloyd‐Jones, 2017). Endothelial dysfunction permits the retention of plasma lipoproteins in the subendothelial space, inducing immune cell infiltration and overproduction of chemokines in ECs (Marchio et al., 2019). Endothelial dysfunction can also promote activation of vascular smooth muscle cells, monocyte recruitment, and atherogenesis via caspase‐1 activation (Tabaei & Tabaee, 2019). Upregulation of cell death among ECs and other cells has been observed in human atherosclerotic lesions, especially in advanced plaques (Rust, Hofer, & Schwab, 2018; Theodorou & Boon, 2018). Therefore, there is an urgent necessity to investigate the molecular mechanism about endothelial dysfunction during human atherosclerosis.

CXCR7 is widely located in brain, heart, kidney, and tumor cells (Al‐Toub et al., 2019; Wang, Chen, & Shen, 2018; Zhang, Zhang, et al., 2018). CXCR7 also extensively expresses in the endothelium cells and is increased by hypoxia (Yamada et al., 2015). Global knockdown of CXCR7 aggravates atherosclerotic lesions by reducing the cholesterol uptake in hyperlipidemic ApoE^−/−^ mice (Li et al., 2014). Endothelium‐specific knockout CXCR7 mice cannot survive because of the resulting heart valve malformation (Sierro et al., 2007). After myocardial infarction (MI), endothelial CXCR7 regulates vascular homeostasis, reduces pathological vascular response, and provides cardiac protective function (Hao et al., 2017). Our previous report also indicates that CXCR7 promotes EC proliferation and angiogenesis (Zhang et al., 2017). However, the molecular mechanisms by which CXCR7 acts in the endothelial injury and atherosclerotic lesions remain unknown.

Recently, cell pyroptosis has been recognized as a new form of cell death in addition to cell apoptosis and necrosis. Pyroptosis is a unique form of cell death mediated by inflammatory response via activation of inflammasome signals (Liu, Zeng, Li, Mehta, & Wang, 2018; Robinson et al., 2019; Zeng, Wang, & Tan, 2019; Zhou et al., 2018). Inflammasome, as a receptor of innate immune, can recognize the pathogen‐associated molecular patterns (PAMPs) and danger‐associated molecular patterns (DAMPs), thus activating downstream signaling cascades, such as caspase‐1 and interleukin‐1β (IL‐1β) (Liston & Masters, 2017; Mehto et al., 2019). Prominent inflammasomes contain NOD‐like receptor family protein (such as NLRP3, NLRP1, NLRC4), the adaptor protein ASC, absent in melanoma 2 (AIM2), and pyrin. Pyroptosis participates in ox‐LDL‐induced human macrophages and EC dysfunction, indicating a critical effect of pyroptosis on atherosclerosis development (Hoseini et al., 2018; Zhaolin et al., 2019; Zi et al., 2019). Activation of NOD‐like receptor family pyrin domain‐containing 3 (NLRP3) increases the atherosclerotic lesions in ApoE^−/−^ mice (Wang, Wu, et al., 2018; Zhuang et al., 2019). Ox‐LDL exposure augments atherosclerotic lesions by enhancing the production of pro‐inflammatory cytokines, including IL‐1β and TNF‐α in macrophages (Hosseini et al., 2015). It is therefore speculated that EC dysfunction is associated with pyroptosis signaling. Nevertheless, whether pyroptosis is involved in EC dysfunction after ox‐LDL exposure and how it is associated with the abnormal generation of inflammatory cytokines still need to be illuminated.

Here, our study explored the potential function of CXCR7 on the ApoE^−/−^ mice fed with HFD and ox‐LDL‐treated HUVECs and the underlying mechanisms. Our results indicated that CXCR7 plays a key role in mediating EC pyroptosis. Therefore, modulation of CXCR7 could have therapeutic benefits for atherosclerosis patients.

## 2 MATERIALS AND METHODS

### 2.1 Human sample collection

Human carotid atherosclerotic plaque samples (*n* = 6) and matched adjacent normal aorta tissue samples from Shanghai Tenth People's Hospital were used to detect the expression of CXCR7 via Western blotting analysis (*n* = 3) and immunofluorescence analysis (*n* = 3). Research protocols were approved by the Clinical Research Ethics Committees of Tongji University, and all patients signed written consent for the use of their samples for biomedical research.

### 2.2 Atherosclerotic mouse model

Twenty‐one male ApoE^−/−^ mice (16–21 g) from the Model Animal Research Center of Nanjing University were housed in standard cages with 20–23°C and 50%–60% humidity. The mice were randomly divided into a normal‐diet group (Control, *n* = 7) and HFD group (Research Diets, D12109 = 40 kcal% fat, 1.25% cholesterol, 0.5% cholic acid added to a basal diet, *n* = 14) for 3 months to create the atherosclerosis model. Then, the HFD group was given normal saline (NS, *n* = 7) or CXCR7 agonist TC14012 (10 mg/kg body weight, *n* = 7) every 6 days for 1 month. After treatment, all mice were euthanized, and the common carotid arteries and aortas were harvested for the evaluation of the aortic lesion and biochemical investigation. Our experiment protocols were developed based on standards of laboratory animal operations formulated by the Animal Ethics Committee of Tongji University and were approved by the Animal Ethics Committee of Tongji University.

### 2.3 Oil red O staining

The lipid deposition in the mouse aorta was measured by Oil Red O staining. Tissues (10 μm) were dissected with deleted fatty deposits stripped by a dissection microscope. After a 2‐min rinse with propylene glycol, sections were stained with Oil Red O dyeing solution for 10 min and then differentiated and rinsed with 85% propylene glycol. Slides were then incubated in hematoxylin for 1 min. Slides were rinsed thoroughly in ddH_2_O. Cover glasses were mounted with aqueous mounting medium (Sigma‐Aldrich). Finally, the images were observed under an optical microscope (Leica Co., Ltd.).

### 2.4 Immunofluorescence analysis

Immunofluorescence staining was used to determine the location of CXCR7. Briefly, the tissue sections were fixed with 4% paraformaldehyde for 30 min. After treatment with 0.6% Triton X‐100 for 1 hr, the sections were blocked with donkey serum. Then, the sections were incubated overnight at 4°C with primary antibodies including anti‐CXCR7 (1:200, Abcam, ab137485) and anti‐vWF (1:200, Abcam, ab154193), followed by incubating with Alexa Fluor 488‐ or 568‐conjugated secondary antibody was added (1:500, Life Technologies). Samples were then co‐stained with DAPI (Sigma‐Aldrich) for 15 min at room temperature. Cover glasses were mounted with aqueous mounting medium (Sigma‐Aldrich, F4680). The images were obtained using a Zeiss Live Cell Imaging System.

### 2.5 Cell culture and transfection

HUVECs were cultured in endothelial cell growth factors with endothelial cell media (ECM) from ScienCell Research Laboratories (ScienCell) at 37°C with 5% CO_2_ incubation. The pCDNA‐CXCR7, shRNA of CXCR7, and the negative controls were synthesized using GenePharma. Cells were transfected using Lipofectamine 2000 reagent (Invitrogen) in 5% CO_2_ incubator at 37°C according to the manufacturer's instructions. After 48 hr, the transfected cells were harvested for Western blot analysis to verify the efficiency of transfection. Then, the transfected HUVECs were treated with ox‐LDL (100 μg/ml), TC14012 (0–30 μM), or both for 24 hr.

### 2.6 Endothelial cell migration assay

EC migration was analyzed using a Transwell migration assay. In brief, HUVECs (5 × 10^4^ cells/well) in 100 μl of endothelial cell media (ECM) were plated into the upper of a modified Boyden chamber, and 15% FBS with 500 μl of fresh basic ECM was added into the down well as a chemoattractant. After incubation at 37°C for 12 hr, the nonmigrating cells were gently took away by a cotton swab, and the migrated cells were dyed with Calcein‐AM at 37°C for 20 min in the dark and analyzed on a microscope in at least six representative fields.

### 2.7 Endothelial cell capillary‐like tube formation assay

HUVECs (2 × 10^5^ cells/well) were seeded in 96‐well plates that had been pre‐coated with Matrigel (80 μl/well, BD Biosciences, USA) for 1 hr. After incubation for 24 hr, images of tube morphology were obtained using an inverted Olympus microscope, and the number of capillary‐like tubes was determined by calculating the quantity of meshes in at least three random microscopic fields.

### 2.8 Western blotting analysis

HUVECs were added to lysis buffer (Thermo Scientific, 78501) with protease inhibitors according to manufacturer's instructions. Equal amounts of total protein (30 μg) were separated by 10% SDS‐PAGE and transferred to PVDF membranes (Millipore) using electroblotting. After blocked with 5% nonfat milk for 1 hr at room temperature, the membranes were incubated with the primary antibodies, including Angiogenesis Antibody Sampler Kit (1:1000, CST, 8696), Inflammasome Antibody Sampler Kit (1:1000, CST, 32961), Cxcr7 (1:1000, Abcam, ab137485), and Cxcr4 (1:1000, Abcam, ab124824) overnight at 4°C, and then incubated with HRP‐conjugated secondary antibodies from Jackson ImmunoResearch (1:5000, AB_2313567) or ICLLAB (1:5000, GGHL‐90P) at room temperature for 1 hr. An enhanced chemiluminescence system (Bio‐Rad) was used to visualize the protein bands.

### 2.9 Statistical analysis

All results are presented as mean ± standard deviation (*SD*) and were analyzed with GraphPad Prism 5. Comparisons were performed with one‐way ANOVA followed by Bonferroni post hoc test. Statistical significance was described as *p* < 0.05.

## 3 RESULTS

### 3.1 CXCR7 expression is upregulated in human atherosclerotic plaque

**FIGURE 1:**
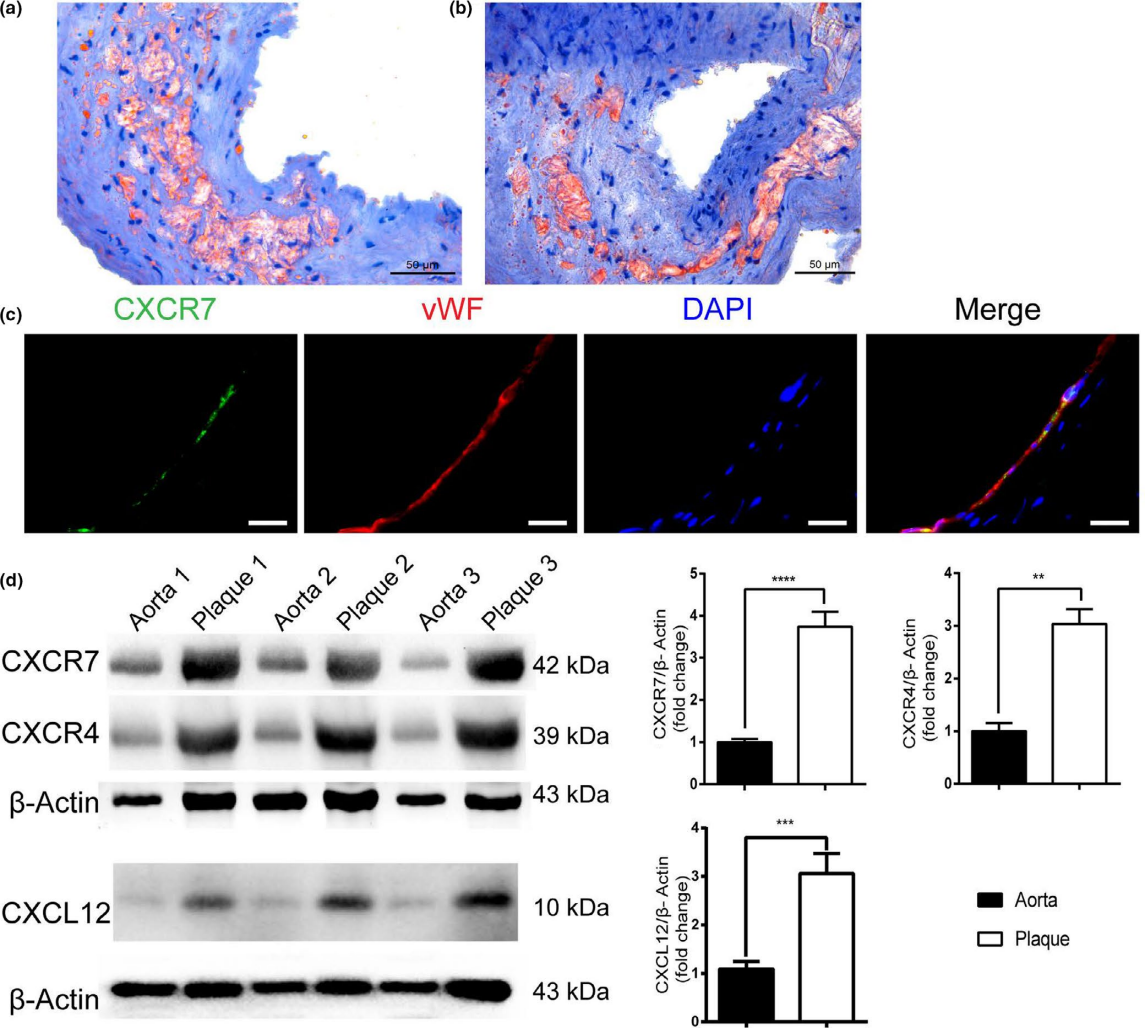
CXCR7 is upregulated in human carotid atherosclerotic plaque tissues. (a, b) Lipid deposition in human carotid atherosclerotic plaque tissues was observed by Oil Red O staining. Bar: 50 μm. (c) Immunofluorescence directly described the expression of CXCR7, vWF, and DAPI in human carotid atherosclerotic plaque tissues. Bar:  20 μm. (d) The expression of CXCR7, CXCL12, and CXCR4 in human carotid atherosclerotic plaque tissues was determined by Western blot analysis. **p < 0.01, ***p < 0.001, ****p < 0.0001 versus the control group; the measurement data are here expressed as mean  ± standard deviation

To explore the potential link between CXCR7 and atherosclerotic lesions, we first evaluated the expression levels of CXCR7 in the carotid atherosclerotic plaque and their matched adjacent normal artery tissue from patients by staining with Oil Red O. Results indicated the presence of atherosclerotic lesions in the carotid atherosclerotic plaque from patients (Figure 1a,b). Furthermore, location patterns of CXCR7 in the plaque were detected by immunofluorescence. The immunofluorescence for CXCR7 and von Willebrand factor (vWF) was colocalized in human carotid atherosclerotic plaque. In this way, CXCR7 was mainly located in the endothelial layer (Figure 1c). Western blot analysis was used to further assess the expression pattern of CXCR7, CXCR4, and CXCL12 in plaque tissues. The data revealed that CXCR7, CXCR4, and CXCL12 protein levels in carotid atherosclerosis plaque tissues were notably elevated compared with their matched adjacent normal aorta tissues (Figure 1d). These data could supply an indication for a potential function that CXCR7 involved in the development of human carotid atherosclerotic plaque.

### 3.2 CXCR7 expression is upregulated in the aortas of HFD‐FED ApoE^−/−^ mice

**FIGURE 2:**
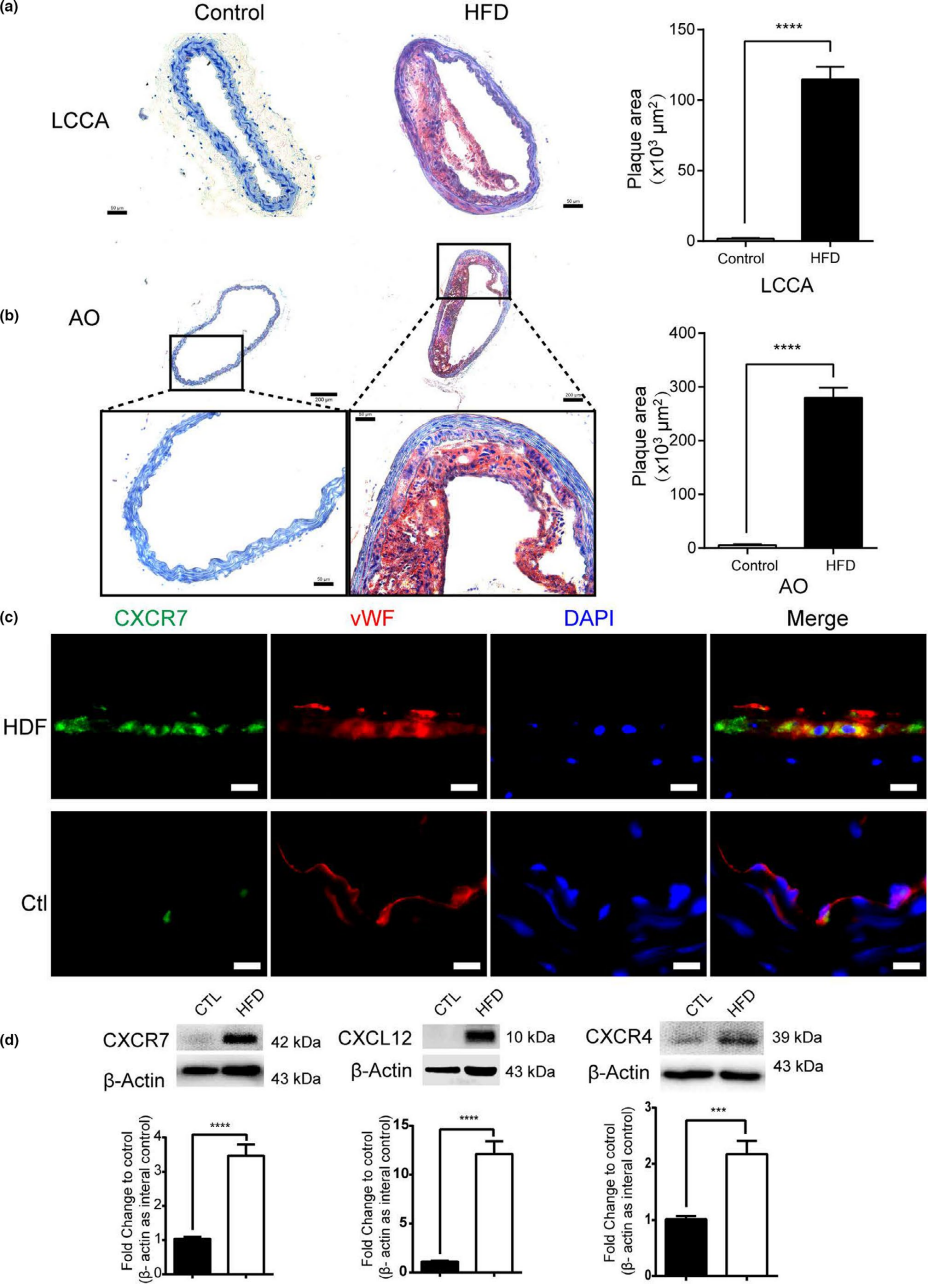
CXCR7 is upregulated in the LCCA of HFD‐treated ApoE^−/−^ mice. (a, b) Lipid deposition in the left common carotid artery (LCCA) and aorta (AO) of ApoE^−/−^ mice was observed by Oil Red O staining. (a) Bar: 50 μm. (b) upper panel bar: 200 μm and bottom panel: 50 μm. (c) Immunofluorescence directly described the expression of CXCR7, vWF, and DAPI in the LCCA of ApoE^−/−^ mice. Bar: 20 μm. (d) The levels of expression of CXCR7, CXCL12, and CXCR4 in the LCCA of ApoE^−/−^ mice and HFD‐treated ApoE^−/−^ mice were determined by Western blot analysis. ***p < 0.001 versus the control group; the measurement data are expressed as mean  ± standard deviation

To investigate the role of CXCR7 during the progression of atherosclerosis, we performed Oil Red O staining in the left common carotid artery (LCCA) of the ApoE^−/−^ mouse tissue sections. Our results showed that high‐cholesterol, high‐fat diet (HFD) stimulated atherosclerotic lesions in the ApoE^−/−^ mice (Figure 2a,b). The locations of CXCR7 in the aorta of the ApoE^−/−^ mice were determined by immunofluorescence assay. Double staining showed the presence of CXCR7 and vWF in the aortas of the ApoE^−/−^ mice. CXCR7 is mainly distributed in the endothelial layer of the ApoE^−/−^ mouse aorta (Figure 2c). Western blot analysis was used to further determine the pattern of expression of CXCR7, CXCR4, and CXCL12 in the aorta of ApoE^−/−^ mice. The data indicated that CXCR7, CXCR4, and CXCL12 protein levels in the aorta of the ApoE^−/−^ mice with HFD were markedly higher than those in the corresponding control group (Figure 2d). These data indicate a functional role of CXCR7 in atherosclerotic lesions of the ApoE^−/−^ mice.

### 3.3 CXCR7 regulates ox‐LDL‐inhibited HUVEC migration and tube formation

**FIGURE 3:**
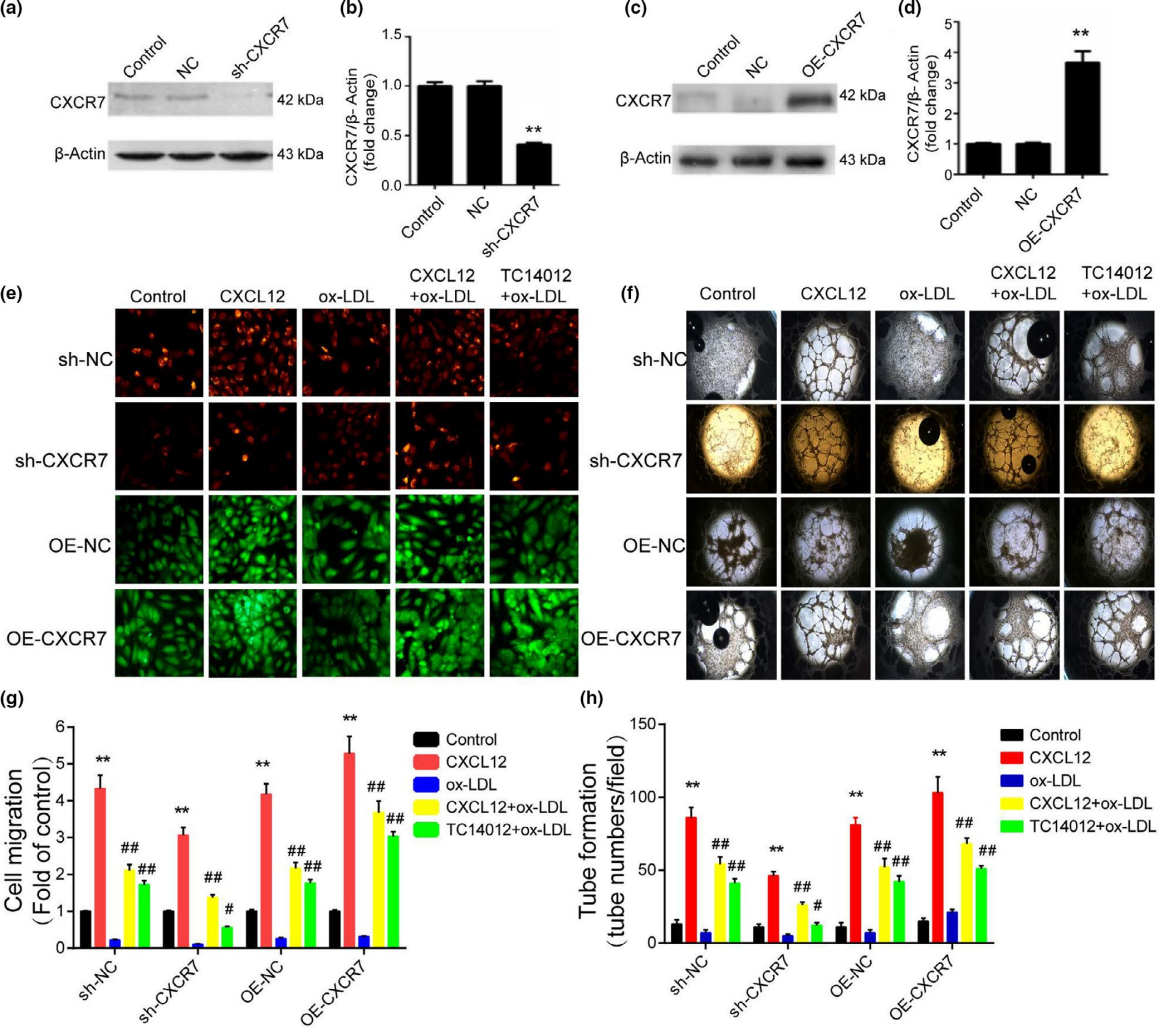
CXCR7 is upregulated in ox‐LDL‐treated HUVECs and mediates ox‐LDL‐induced endothelial injury. (a, b, c, d) HUVECs were transfected with sh‐NC, sh‐CXCR7, pcDNA‐NC, and pcDNA‐CXCR7, respectively. Then, CXCR7 expression in the HUVECs after transfection was determined by Western blot analysis. (e, g) HUVECs were transfected with sh‐NC, sh‐CXCR7, pcDNA‐NC, and pcDNA‐CXCR7, respectively. Then, HUVECs were treated with ox‐LDL (100 μg/ml) with CXCL12 (100 ng/ml) or TC14012 (30 nM) for 12 hr. After that, HUVECs migration were assessed by Transwell assay. Data are representative of six random microscopic field images taken at 100× magnification. (f, h) HUVECs were transfected with sh‐NC, sh‐CXCR7, pcDNA‐NC, and pcDNA‐CXCR7. Then, HUVECs were treated with ox‐LDL (100 μg/ml) with CXCL12 (100 ng/ml) or TC14012 (30 nM) for 24 hr. Then, HUVEC angiogenesis was assessed by tube formation assay. Data are representative of six random microscopic field images taken at 20× magnification. **p < 0.01 versus the control group; the measurement data are expressed as mean  ± standard deviation

We used the loss‐of‐function and gain‐of‐function approaches to investigate the essential role of CXCR7 in triggering HUVEC migration and tube formation. CXCR7 was knocked down by transfecting CXCR7 shRNA into HUVECs, and the knockdown was confirmed (Figure 3a,b). CXCR7 was overexpressed by transfecting pCDNA‐CXCR7 into HUVECs, and overexpression was also confirmed (Figure 3c,d). The biological behavior of HUVECs was investigated. Transwell chamber assay indicated that knockdown of CXCR7 reduced the migration rate of HUVECs (Figure 3e,g), in contrast to that overexpression of CXCR7 induced the migration rate of HUVECs (Figure 3e,g). Ox‐LDL significantly reduced the migration rate of HUVECs. The optimum concentration of ox‐LDL was found to be 100 µg/ml. We used this concentration, 100 µg/ml, of ox‐LDL for the experiments described below. We then used a Transwell chamber assay to investigate how CXCR7 affects the migration behavior of ox‐LDL‐treated HUVECs. Knockdown of CXCR7 decreased the migration rate of ox‐LDL‐treated HUVECs, while CXCL12 and TC14012 were each found to partly reverse this effect (Figure 3e,g). Overexpression of CXCR7 significantly increased the migration rate of ox‐LDL‐treated HUVECs, and CXCL12 and TC14012 were each able to enhance this effect (Figure 3e,g).

Next, we performed a tube formation assay to assess the effect of CXCR7 on HUVECs' tube formation ability. As in the migration assay results, the data indicated that knockdown of CXCR7 reduced the tube formation ability of HUVECs (Figure 3f,h); however, overexpression of CXCR7 induced the tube formation ability of HUVECs (Figure 3f,h). Ox‐LDL significantly reduced the tube formation ability of HUVECs. We also used a tube formation assay to investigate how CXCR7 affects ox‐LDL‐treated HUVECs. Knockdown of CXCR7 decreased the tube formation ability of ox‐LDL‐treated HUVECs. CXCL12 and TC14012 were each able to partly reverse this effect (Figure 3f,h). Overexpression of CXCR7 significantly improved the decreased tube formation ability of ox‐LDL‐treated HUVECs, and CXCL12 and TC14012 were each found to render this improvement more pronounced (Figure 3f,h). Collectively, these results indicated that CXCR7 mediated ox‐LDL‐treated HUVEC migration and tube formation.

### 3.4 Cell pyroptosis increases in human atherosclerotic plaque and the LCCA of HFD‐FED ApoE^−/−^ mice

**FIGURE 4:**
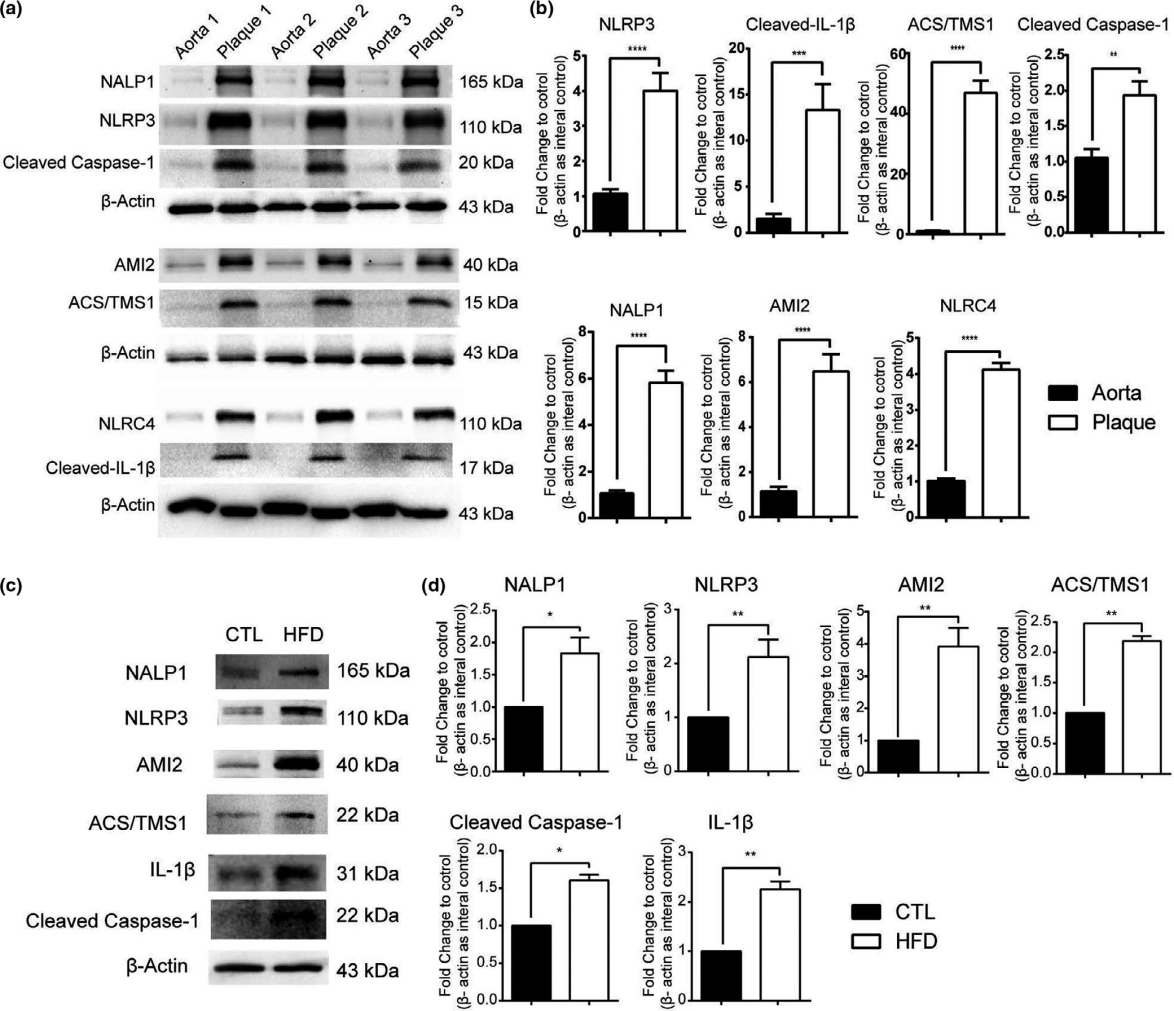
The pyroptosis marker was detected in human carotid atherosclerotic plaque tissues, the LCCA of HFD‐induced ApoE^−/−^ mice, and ox‐LDL‐treated HUVECs. (a, b) The pyroptosis signal in human carotid atherosclerotic plaque tissues was assessed by Western blot analysis. (c, d) The pyroptosis signal was assessed in the LCCA of HFD‐induced ApoE^−/−^ mice by Western blot analysis. *p < 0.05, **p < 0.01, ***p < 0.001, ****p < 0.0001 versus the control group; the measurement data are here expressed as mean  ± standard deviation

A Western blot assay was performed to characterize the expression of pyroptosis‐related proteins in human carotid atherosclerotic plaque and their matched adjacent normal aorta tissues. The results showed that the expression levels of NALP1, NLRP3, AIM2, ASC/TMS1, caspase‐1, IL‐1β, and NLRC4 were significantly increased in human carotid atherosclerotic plaque tissues compared with those in the matched adjacent normal aorta tissues (Figure 4a,b). Next, we used Western blot analysis to determine whether the expression of pyroptosis‐related proteins was altered in the left common carotid artery (LCCA) of HFD‐fed ApoE^−/−^ mice. The results shown in Figure 4c,d showed that the expression of NALP1, NLRP3, AIM2, ASC/TMS1, caspase‐1, IL‐1β, and NLRC4 was significantly increased in the LCCA of HFD‐fed ApoE^−/−^ mice, indicating that the rate of cell pyroptosis increases in human carotid atherosclerotic plaque and the LCCA of HFD‐fed ApoE^−/−^ mice.

### 3.5 CXCR7 mediates cell pyroptosis in ox‐LDL‐injured HUVECs

**FIGURE 5:**
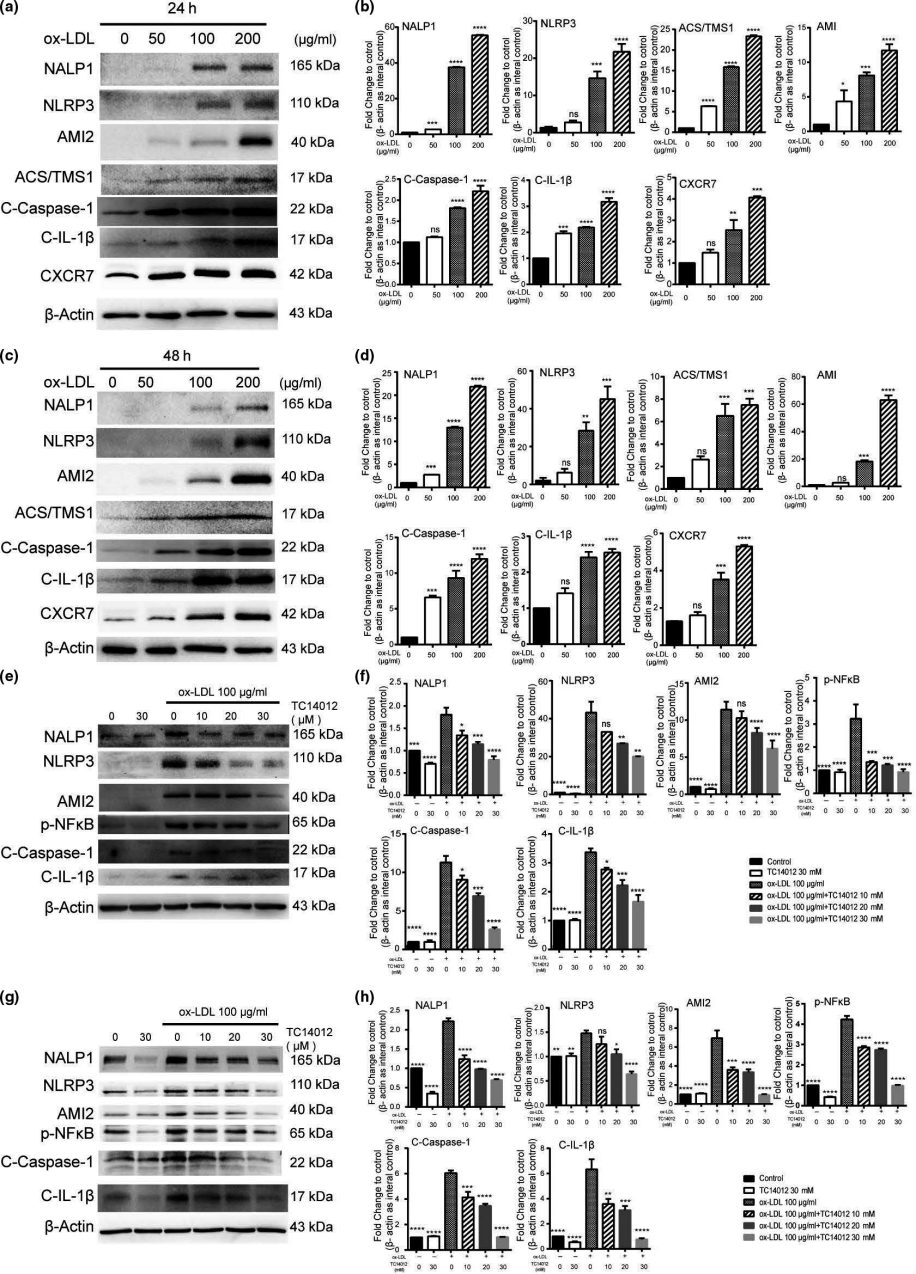
Upregulation of CXCR7 decreases pyroptosis in HUVECs. (a, b) HUVECs were treated with ox‐LDL (100–200 μg/ml) for 24 hr. Then, the pyroptosis signal was detected by Western blot analysis. (c, d) HUVECs were treated with ox‐LDL (100–200 μg/ml) for 48 hr. After that, the pyroptosis signal was detected by Western blot analysis. (e–h) HUVECs were transfected with sh‐NC or sh‐CXCR7, respectively. After that, the pyroptosis signal was detected by Western blot analysis. *p < 0.05, **p < 0.01, ***p < 0.001, ****p < 0.0001 versus the control group; the measurement data are here expressed as mean ± standard deviation

We performed *in vitro* investigations using ox‐LDL‐treated HUVECs to elucidate the association between ox‐LDL and EC pyroptosis. HUVECs were cultured with different concentrations of ox‐LDL (0, 50, 100, and 200 µg/ml) for 24 and 48 hr, and the levels of expression of CXCR7, NALP1, NLRP3, AIM2, ASC/TMS1, caspase‐1, and IL‐1β were significantly increased in a dose‐ and a time‐dependent manner (Figure 5a–d). We also investigated how CXCR7 affects ox‐LDL‐induced HUVEC pyroptosis. HUVECs were pretreated with TC14012 (10, 20, 30 µM) and then added with ox‐LDL (100 µg/ml) for 24 and 48 hr. The Western blot results showed that ox‐LDL induced HUVEC pyroptosis. TC14012 reversed this effect in a dose‐ and a time‐dependent manner (Figure 5e–h).Our results suggested that CXCR7 may be involved in ox‐LDL‐induced HUVEC pyroptosis.

### 3.6 CXCR7 mediates cell pyroptosis in the LCCA of HFD‐FED ApoE^−/−^ mice

**FIGURE 6:**
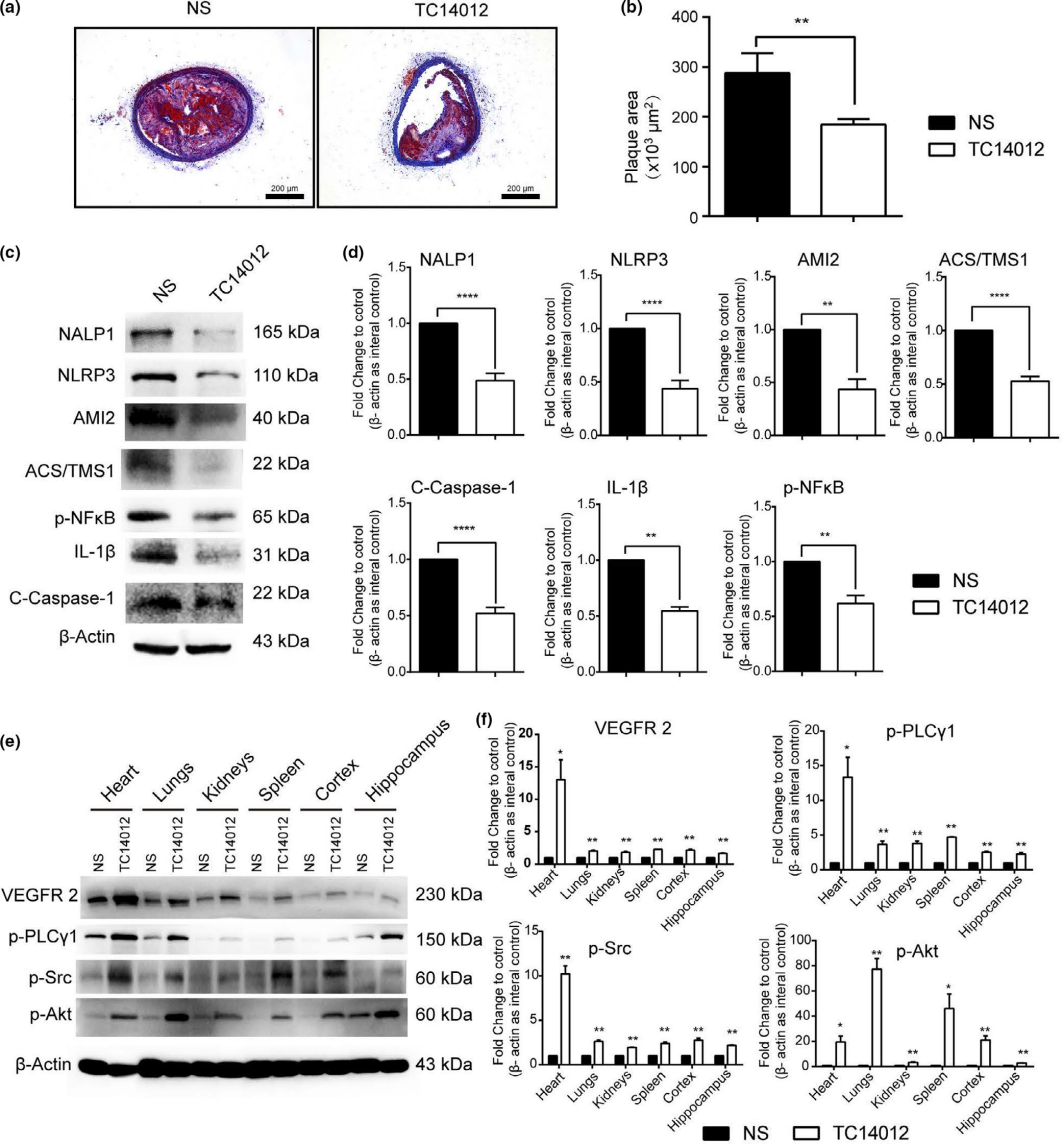
Activation of CXCR7 reduces endothelial injury via mitigating the pyroptosis pathway and increasing angiogenesis signal *in vivo*. (a, b) After treatment with normal saline (NS) or TC14012 for 4 weeks, the atherosclerotic lesion area in the left common carotid artery (LCCA)of HFD‐induced ApoE^−/−^ mice was determined by Oil Red O staining; bar: 200 μm. (c, d) After treatment with NS or TC14012 for 4 weeks, the pyroptosis signal was detected in the LCCA of ApoE^−/−^ mice; (e, f) After treatment with NS or TC14012 for 4 weeks, the angiogenesis pathway was assessed in the mouse tissues. *p < 0.05, **p < 0.01, ***p < 0.001, ****p < 0.0001 versus the control group; the measurement data are here expressed as mean ± standard deviation

To dissect the role of CXCR7 during the progression of atherosclerosis *in vivo*, we performed Oil Red O staining on sections of LCCA from HFD‐fed ApoE^−/−^ mice. Our results showed that TC14012 treatment for 1 month reduced atherosclerotic lesions in the LCCA of the HFD‐fed ApoE^−/−^ mice (Figure 6a,b). Western blot results showed that the induction of cell pyroptosis in the LCCA of the HFD‐treated ApoE^−/−^ mice was reversed by TC14012 treatment (Figure 6c,d). The expression of angiogenesis‐related proteins, such as VEGFR2, p‐Akt, p‐PLC, and p‐SRC, was higher in HFD‐fed ApoE^−/−^ mice given TC14012 pretreatment (Figure 6e,f). These data suggest CXCR7 might be involved in cell pyroptosis and angiogenesis in HFD‐fed ApoE^−/−^ mice *in vivo*.

## 4 DISCUSSION

Atherosclerosis has been widely recognized as the main cause of myocardial infarction and stroke (GBD 2016 Lifetime Risk of Stroke Collaborators, 1990; Malik, 2018). It has been indicated that reduction of endothelial cell pyroptosis prevents the inflammatory response, thus reducing the atherosclerotic lesions (Liu et al., 2017; Zhang, Liu, et al., 2018). However, effective therapy for atherosclerosis can be inadequate, because the mechanisms associated with cell pyroptosis are still unknown. Specifically, the mechanisms by which ox‐LDL promotes atherosclerosis via cell pyroptosis signal are ambiguous. In view of this, *in vitro* and *in vivo* atherosclerosis models were here established to explore the effect of endothelial cell pyroptosis on atherosclerosis progression in this study. Our data exhibited that CXCR7 is upregulated in human carotid plaque tissues, HFD‐fed ApoE^−/−^ mouse aortas, and ox‐LDL‐treated HUVECs. Moreover, activation of CXCR7 alleviates ox‐LDL‐induced endothelial injury and cell pyroptosis. In addition, the activation of CXCR7 reduces the size of atherosclerotic lesions by mitigating the pyroptosis pathway. Therefore, activation of CXCR7 reduces atherosclerosis progression by mitigating the pyroptosis pathway *in vivo* and *in vitro*.

CXCR7 provokes the proliferation of EC and ischemia‐stimulated angiogenesis. Our previous report also showed that the CXCL12/CXCR7 axis mediates angiogenesis, and CXCR7 may be a target for ischemic disease treatment (Zhang et al., 2017). Other studies indicate that endothelial CXCR7 serves as a key organizer of vascular function and progress, and it has the ability to regulate cardiac function (Hao et al., 2017). More importantly, endothelial cells can trigger the directing signals that control the formation of atherosclerotic lesions. Thus, endothelial dysfunction of atherosclerosis is considered an initiating factor in the process of atherosclerosis (Bibli et al., 2019; Siasos et al., 2018). Nevertheless, whether CXCR7 is involved in endothelial dysfunction in atherosclerosis is still obscure. In this study, our findings showed that CXCR7 was far more highly expressed in plaque tissues than non‐plaque tissues from patients with carotid atherosclerosis, ApoE^−/−^ mice fed with HFD, and ox‐LDL‐treated HUVECs than in the controls (Figures 1, 2 and 5). So, CXCR7 may be involved in atherosclerosis development *in vivo* and *in vitro*. Next, we found that the upregulation of CXCR7 reversed ox‐LDL injury to EC function, such as migration and tube formation, through loss‐of‐function and gain‐of‐function assays (Figure 3). These results suggest that CXCR7 overexpression can suppress atherosclerotic lesion formation. Nevertheless, how CXCR7 plays a protective role against endothelial injury in atherosclerosis progression is still unknown.

Here, we suspected that CXCR7 may be involved in endothelial pyroptosis and atherosclerosis progression. Cell pyroptosis is a new form of cell death, which has been recognized recently (Liu et al., 2018; Robinson et al., 2019; Zeng et al., 2019; Zhou et al., 2018). It is mediated by inflammatory response via activation of inflammasome signals. Cell pyroptosis is dependent on the activation of caspase‐1, which can stimulate cytokine IL‐1β into the active forms and then induce pyroptotic cell death. Recent studies indicated that reduction of endothelial cell pyroptosis prevents the inflammatory response, thus reducing the atherosclerotic lesions (Liu et al., 2017; Zhang, Liu, et al., 2018). Correspondingly, results from a large clinical trial verified that the risk of recurrent cardiovascular events has been significantly reduced after treatment with an anti‐IL‐1β antibody (Ridker et al., 2017). These studies indicated that target immunity and inflammatory response might be a new strategy to counteract atherosclerosis (Zhao & Mallat, 2019). Abnormal cellular innate immune response and oxidative stress triggered cell death, including cell pyroptosis. Moreover, it has been well known that endothelial cells are the key cell type to be damaged by ox‐LDL and other inflammatory cytokines (Shan et al., 2016). Consequently, inhibiting cell pyroptosis of ECs might efficiently limit the progression of atherosclerosis, but the underlying mechanism is still unclear (Hoseini et al., 2018; Zi et al., 2019). In this study, we found that the cells displayed specific features of pyroptosis in plaque tissues from patients with carotid atherosclerosis and the aorta of ApoE^−/−^ mice fed with HFD, as shown by upregulated levels of NALP1, NLRP3, AIM2, ASC/TMS1, caspase‐1, and IL‐1β, indicating that induction of cell pyroptosis might be a cellular mechanism underlying the progressions of atherosclerosis (Figure 4). Hence, we highlight the importance of regulating the expression of cell pyroptosis signals in ECs to essentially decelerate atherosclerosis.

The former data showed that cell pyroptosis signals are involved in EC function. Next, we explored the protective role of CXCR7 in EC treated with ox‐LDL via the expression of cell pyroptosis signal. We here found that the expression of CXCR7, NALP1, NLRP3, AIM2, ASC/TMS1, caspase‐1, and IL‐1β was upregulated in HUVECs treated with ox‐LDL compared with the control group. In contrast, activation of CXCR7 by TC14012 in HUVECs partly reversed ox‐LDL‐induced pyroptotic death (Figure 5). Based on this evidence, we here suggested that CXCR7 is involved in ECs' pyroptosis and atherosclerosis *in vitro*.

Consistent with the data from our *in vitro* researches, we found that activation of CXCR7 by TC14012 reduces atherosclerotic lesions in HFD‐treated ApoE^−/−^ mice (Figure 6). Furthermore, reduction of atherosclerotic lesions in the LCCA of HFD‐fed ApoE^−/−^ mice correlated with decreased cell pyroptosis. The present work showed for the first time that TC14012 contributed to reductions in atherosclerotic lesions, at least in part, by mitigating pyroptosis and inducing the angiogenic pathway *in vivo*.

EC dysfunction is known as the initial event that mediated atherosclerotic lesions. Previous studies revealed that before cholesterol crystal formatted plaque in the vascular, ECs preferentially initiate cell pyroptosis leading to endothelial dysfunction. Herein, we present that TC14012 treatment for 1 month reduced atherosclerotic lesions in the LCCA of the HFD‐fed ApoE^−/−^ mice (Figure 6a,b), as well as the cell pyroptosis in the LCCA (Figure 6c,d). However, the plaque burden in the aorta still remains unchanged (Figure S1). Therefore, it is possible that CXCR7 agonist treatment prior to contributes to the reduction of cell pyroptosis. It also has been reported that the prevalence of atherosclerosis in the carotids is higher than that in the aorta (Fernandez‐Friera et al., 2015). Moreover, the highly inflamed plaques have a larger size and are more unstable (Fernandez‐Friera et al., 2019; Hansson, Libby, & Tabas, 2015). Emerging evidences indicate that carotid artery plaques are more relevant to the extent of vascular inflammation than aorta by using the imaging detecting technology (Fernandez‐Friera et al., 2019). So, these findings may, at least to some extent, help to explain and support our results.

Consistent with the notion that CXCR7 agonist treatment inhibits cell pyroptosis, the regulation of blood lipid levels, including triglyceride, TC, HDL‐C, and LDL‐C levels in the mouse models, should be analyzed in further studies. The current study did not find evidence that if plaque burden changed in the aorta, the expression of pyroptosis signal proteins was reduced (Figure S1). Subsequently, the effect of long‐time treatment of CXCR7 agonist on plaque burden should be further researched.

Another key problem about atherosclerotic lesions development is accompanied by an altered plaque composition. Recent experimental data support that monocyte/macrophage accumulation and collagen degradation mediate atherosclerotic plaque formation. Nonetheless, the atherosclerotic lesions are deemed more complicated, and plaque composition parameters were not measured in this study. So, the effect of CXCR7 on plaque composition and stability still remains uncertain.

The complicated processions through the formation of atherosclerotic lesions need the connection between EC and smooth muscle cells (VSMC), and communicate with the surrounding microenvironment. Further study exploring the mechanisms about the formation of atherosclerotic lesions, especially about the involvement of VSMC in atherosclerosis, is needed. Besides, atherosclerosis is a chronic inflammatory disease (Gistera & Hansson, 2017). In this study, we treated HUVECs with ox‐LDL for 24 and 48 hr. Although it is widely accepted for the study of atherosclerosis *in vitro*, it may not fully reflect the chronic inflammation response in patients who have atherosclerosis for many years. Moreover, the precise mechanism of CXCR7 on cell pyroptosis remains incompletely understood; the downstream regulatory proteins between CXCR7 and NLRP3 inflammasome pathway require further elucidation.

Collectively, our results provide the first evidence that CXCR7 upregulation reversed ox‐LDL stimulated NLRP3 and caspase‐1 activity and ultimately induced EC pyroptosis *in vitro*. Further, inhibition of endothelial pyroptosis is likely a cellular mechanism underlying the effect of CXCR7 activation on atherosclerotic lesions via regulation of NLRP3/caspase‐1 pathway. Targeting CXCR7‐mediated cell pyroptosis might be a suitable new approach for alleviating atherosclerotic lesions.

## CONFLICT OF INTEREST

The authors have no conflicts of interest to report.

## AUTHOR CONTRIBUTIONS

Lisha Qiu and Min Zhang designed and performed experiments and wrote the manuscript. Sheng Zhang and Yalin Tang participated in human and mouse sample collection and immunohistochemistry experiments. Yanyan Zhang and Congcong Li analyzed data and generated figures. Yi Wang helped with editing and writing. Li Jiang and Jialin C. Zheng designed experiments and participated in analysis of all data and writing of the manuscript.

## Data Availability

All data required to reproduce these findings are available from the corresponding author upon request.
