# Supplementary material for: Activation of CXCR7 promotes endothelial repair and reduces the carotid atherosclerotic lesions through inhibition of pyroptosis signaling pathways
Source: Aging Cell. 2020 Jul 27;19(9):e13205. doi: 10.1111/acel.13205 (PMC7511884; doi:10.1111/acel.13205)
Supplement: Supplementary file 1 [file ACEL-19-e13205-s001.docx]

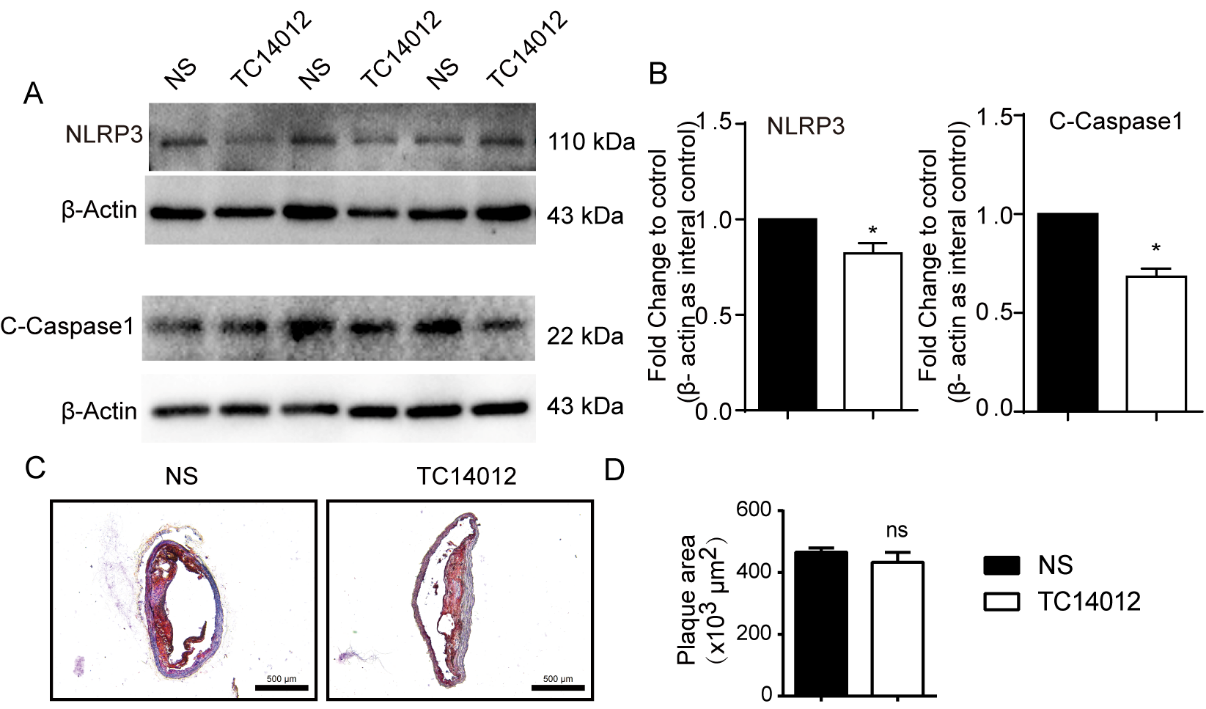


Supplement Fig. 1 Activation of CXCR7 reduces endothelial injury via mitigating the pyroptosis pathway *in vivo*. (A, B) After treatment with Normal saline (NS) or TC14012 for 4 weeks, the atherosclerotic lesion area in the aortas (AO) of HFD-induced ApoE−/− mice were determined by oil red O staining; AO bar: 500 μm . (C, D) After treatment with NS or TC14012 for 4 weeks, the pyroptosis signal was detected in the aortas of ApoE−/− mice.
